# Heterotrimeric G Protein–RasGAP Coupling Drives Adaptation During Chemotaxis

**DOI:** 10.3390/cells15090819

**Published:** 2026-04-30

**Authors:** Xuehua Xu, Riley D. Kim, Haneul Hyun, Ranti Dev Shukla, Tian Jin

**Affiliations:** Chemotaxis Signaling Section, Laboratory of Immunogenetics, National Institute of Allergy and Infectious Diseases, National Institutes of Health, 5625 Fishers Lane, Rockville, MD 20850, USA; rileykim854@gmail.com (R.D.K.); rantibio2@gmail.com (R.D.S.);

**Keywords:** chemotaxis, G protein-coupled receptor (GPCR), heterotrimeric G protein, adaptation, Ras GTPase activating protein (RasGAP)

## Abstract

**Highlights:**

Chemotaxis, the directional migration of cells along chemoattractant gradients, underlies processes such as neuron patterning, lymphocyte recruitment, cancer metastasis, and Dictyostelium discoideum development. The hallmark of eukaryotic chemotaxis is the ability to sense and respond to gradients spanning wide concentration ranges through cellular adaptation. This process involves three interconnected modules: gradient sensing, cell polarity, and migration, with gradient sensing serving as the foundation. While many components of G-protein-coupled receptor (GPCR)-mediated signaling are known, the molecular mechanisms driving adaptation remain unclear. Here, we show that the heterotrimeric G protein α subunit interacts with RasGAP C2GAP1 to mediate adaptation through local inhibition during gradient sensing and chemotaxis.

**What are the main findings?**

- C2GAP1 is essential for concentration-dependent adaptation during gradient sensing, builds local inhibition, and facilitates rapid reorientation in dynamic gradients.
- The direct association between C2GAP1 and Gα2, with preferential interaction with activated Gα2, attenuates both Ras activity and heterotrimeric G-protein activation.

**What is the implication of the main finding?**

- Activated Gα2 directly recruits its own negative regulator C2GAP1, forming a self-limiting adaptive feedback circuit.
- The coupling of Gα2 and C2GAP1 in an actin-independent fashion constitutes the core components of adaptation during gradient sensing and chemotaxis to enable signaling amplitude and modulate the dynamic range of gradient sensing.

**Abstract:**

Chemotaxis enables eukaryotic cells to detect and migrate along extracellular chemoattractant gradients spanning several orders of magnitude. This remarkable dynamic range relies on adaptation, a process that allows cells to reset their signaling machinery while preserving sensitivity to incremental changes in stimulus intensity. Although numerous actin-dependent feedback mechanisms have been characterized, the molecular basis of adaptation within an actin-independent core gradient-sensing module remains poorly understood. Here, we identify the Ras GTPase-activating protein, C2GAP1, as a critical F-actin-independent effector of the heterotrimeric G protein, Gα2, in *Dictyostelium discoideum*. Using cytoskeleton-free gradient-sensing cells, quantitative imaging, biochemical assays, FRET-based G-protein activation measurements, and structural modeling, we demonstrate that C2GAP1 controls concentration-dependent adaptation during gradient sensing. Mechanistically, C2GAP1 directly associates with Gα2 in both GDP- and GTP-bound states, with preferential binding to activated Gα2, thereby sustaining membrane recruitment and locally attenuating Ras and downstream signaling. Loss of C2GAP1 enhances G-protein activation, disrupts local inhibition, and impairs rapid reorientation in dynamic gradients. These findings define a direct coupling between heterotrimeric G proteins and the RasGAP, C2GAP1, as a core adaptive module that enables gradient sensing across a wide concentration range.

## 1. Introduction

Chemotaxis is a form of directional cell migration guided by chemoattractant gradients. This cellular behavior play- essential roles in numerous physiological processes, such as neuron patterning, recruitment of lymphocytes, angiogenesis, and metastasis of cancer cells [1,2,3]. The social amoeba *Dictyostelium discoideum* has served as a powerful model system for dissecting the conserved signaling architecture of eukaryotic chemotaxis [4,5]. In this system, chemotactic responses to cyclic AMP (cAMP) are mediated by the GPCR cAMP receptor 1 (cAR1), which couples to the heterotrimeric G protein composed of Gα2 and Gβγ subunits [6]. Upon ligand binding, cAR1 activates heterotrimeric G-proteins through dissociation of Gα2 from Gβγ, thereby initiating downstream signaling cascades that drive directed migration [7,8,9]. The molecular mechanisms governing cAR1/Gα2Gβγ-mediated chemotaxis have been extensively investigated. Downstream of G-protein activation, Ras proteins serve as central signaling nodes that activate multiple pathways—PI3K, TORC2, PLA2, ElmoE, and soluble guanylyl cyclase (sGC)—which together coordinate chemotactic signaling and cell movement [10,11,12,13,14].

Several regulatory mechanisms acting at the level of G proteins have been described. Ligand-induced phosphorylation of cAR1 reduces its coupling to heterotrimeric G proteins and functions as a receptor desensitization mechanism [15,16,17]. G-protein-interacting protein 1 (GIP1) binds Gα2 and facilitates its shuttling between the cytosol and plasma membrane, ensuring sufficient availability of G proteins at the membrane during chemotaxis [18]. ElmoE is an effector of Gβ that associates with Gβ to activate Rac and thereby promote cell migration [14]. In addition, the nonreceptor guanine nucleotide exchange factor (GEF) Ric8 acts as a Gα2 effector to amplify Gα2 signaling [19]. Importantly, chemotaxis is a coordinated process comprising three conceptually distinct but interconnected modules: gradient sensing, cell polarity, and cell migration. Gradient sensing can be uncoupled from both initial polarity establishment and actin-based motility [6]. For example, cells treated with actin polymerization inhibitors lose their filamentous actin-based cytoskeleton and become immobile; nevertheless, they retain the ability to sense a chemoattractant gradient. This observation demonstrates that the core gradient-sensing machinery operates independently of F-actin-based cytoskeletal structures. In contrast, the functions of ElmoE and Ric8 require an intact actin cytoskeleton, indicating that these regulators operate within F-actin-dependent feedback loops rather than within the actin-independent core gradient-sensing module. Thus, despite extensive characterization of actin-dependent signaling pathways, the key components that constitute the actin-independent core machinery responsible for gradient sensing remain largely unidentified and represent a critical gap in our understanding of eukaryotic chemotaxis.

Chemotactic cells detect and respond to an enormous concentration range of chemoattractants. For example, *D. discoideum* cells chemotax toward their chemoattractant cAMP gradients from 10^−9^ to 10^−5^ M [20]. To chemotax through gradients with such a large concentration range, cells employ a mechanism called adaptation [6]. The temporal properties of adaptation were first characterized using uniformly applied chemoattractant stimuli [21,22]. In response to sustained stimuli, cells exhibit a transient signaling response that subsequently returns toward baseline despite the continued presence of the stimulus, a phenomenon of adaptation. A defining feature of adaptation is that cells become insensitive to the persistent stimulus while retaining the ability to respond to further increases in chemoattractant concentration. In *D. discoideum*, cAR1 GPCR (cAMP receptor)-mediated phosphatidylinositol (3,4,5)-trisphosphate (PIP_3_) responses display all hallmark features of chemoattractant sensing and adaptation [6]. When cAMP is applied uniformly, the signaling pathway leading to PIP_3_ production can be divided into four sequential steps with distinct kinetics. First, cAMP binds to the cAR1 receptor [23,24]. Second, activated cAR1 induces a persistent dissociation/activation of heterotrimeric G-proteins (Gα2Gβγ) [7,8], indicating that adaptation occurs downstream of G protein activation. Third, Ras proteins are activated by GEFs, which catalyze the exchange of Ras-GDP for Ras-GTP; Ras-GTP is subsequently inactivated by Ras GTPase-activating proteins (RasGAPs), which stimulate its intrinsic GTPase activity [11,25,26,27]. Uniform cAMP stimulation elicits a transient Ras activation followed by concentration-dependent, imperfect adaptation [28,29]. Fourth, Ras-activated PI_3_K phosphorylates phosphatidylinositol 4,5-bisphosphate (PtdIns(4,5)P2, PIP_2_) to phosphatidylinositol (3,4,5)-trisphosphate (PtdIns(3,4,5)P3, PIP_3_) in the membrane; while the lipid phosphatase PTEN transiently dissociates from the membrane to permit accumulation of PIP_3_ and then returns to dephosphorylate PIP_3_ back to PIP_2_ [12,13]. Collectively, these observations identify Ras activation as the earliest step in the GPCR-mediated signaling cascade that exhibits adaptive behavior.

The *D. discoideum* genome encodes at least 18 putative RasGAP proteins, suggesting extensive regulatory potential at the level of Ras inactivation. To date, two RasGAPs, DdNF1 (*ddnf1*) and C2GAP1 (*c2gapA*), have been shown to play critical roles in cAMP-induced adaptation and chemotaxis [28,30,31]. It is widely believed that chemotaxis across a broad range of chemoattractant concentrations relies on precise spatiotemporal regulation of Ras activity through coordinated adaptation mechanisms. Adaptation is often manifested by cell responses upon uniformly applied chemoattractant stimulation (uniform stimulation) [21,32]. Under uniform stimulation in F-actin-free cells, WT cells exhibit transient Ras activation, whereas *ddnf1^−^* cells show prolonged Ras activation [30]. In response to the same stimulation, *c2gapA^−^* cells also display an initial transient Ras activation similar to WT cells, but fail to maintain adaptation during sustained stimulation [28]. The Ras activation profiles observed in WT, *ddnf1^−^*, and *c2gapA^−^* cells suggest that multiple RasGAP proteins cooperate to mediate Ras adaptation. Specifically, F-actin-independent adaptation of Ras signaling appears to involve two sequential phases: an initial transient activation followed by persistent adaptation. Both DdNF1 and C2GAP1 are required for this adaptive process, but they seem to function at distinct steps. DdNF1 appears to mediate the rapid attenuation of Ras activation during the initial phase, whereas C2GAP1 plays a more prominent role in sustaining long-term adaptation during the later phase, particularly under gradient-sensing conditions. However, the molecular basis by which GPCR signaling controls the spatial and temporal dynamics of RasGAPs for adaptation during gradient sensing remains poorly understood.

In the present study, we characterized the spatiotemporal dynamics of gradient sensing in cytoskeleton-free, gradient-sensing cells in the presence or absence of C2GAP1 (*c2gapA^−^*) in response to steady and dynamically changing gradients. We found that exposure to a chemoattractant gradient induces cAMP concentration-dependent plasma membrane recruitment of C2GAP1, and that *c2gapA^−^* cells are impaired in the high-concentration-dependent adaptation process during gradient sensing. Our data further provide a molecular mechanism in which C2GAP1 interacts with Gα2 to restrain G-protein activation, thereby maintaining cellular sensitivity, and attenuating Ras activation during concentration-dependent adaptation and local inhibition during gradient sensing. Together, these functions enable rapid orientation in steady gradients and efficient reorientation in dynamically changing gradients.

## 2. Materials and Methods

### 2.1. Cell Lines, Cell Growth and Differentiation

*Dictyostelium discoideum* wild-type cells lines were acquired from Dictybase.org. The *c2gapA^−^* cell line was constructed as previously reported [28]. Cell growth, transformation, and differentiation were carried out as previously described [12].

### 2.2. Imaging and Data Processing

Differentiated cells (5 × 10^4^) in DB buffer with 2.5 mM caffeine were plated and allowed to adhere to the cover glass of a 4-well or a 1-well chamber (Nalge Nunc International, Naperville, IL, USA) for 10 min and then covered with DB buffer for the live cell imaging experiment. If necessary, cells were treated with 5.0 μM Lat B (Molecular Probes, Eugene, OR, USA) for 10 min prior to the experiments. Cells were imaged using a Carole Zeiss Laser Scanning Microscope Zen 780 (Carl Zeiss, Thornwood, NY, USA), with a 40x/NA 1.3 Oil DIC Plan-Apochromatic objective. To obtain cytoskeleton-free, immobile cells, cells were treated with 5 μM latrunculin B (Thermo Fisher Scientific Inc., Waltham, MA, USA) at a final concentration at room temperature for 10 min prior to the experiments. To establish a steady gradient for chemotaxis or gradient-sensing measurements, we set the FemtoJet (FemtoJet and micromanipulator 5171, Eppendorf, Germany) with Pc = 70 and Pi = 70 to ensure the injection of a constant and small volume of cAMP and Alexa 594 into a one-well chamber (Thermo Fisher Scientific Inc., Waltham, MA, USA) as previously described [33]. Under this condition, a stable gradient was established within 100 μm around the tip of the micropipette. To visualize the gradient, cAMP was mixed with Alexa 594 (Thermo Fisher Scientific Inc., Waltham, MA, USA) at a final concentration of 0.1 mg/mL. To suddenly expose a cell to a stable gradient or reapplication of the identical gradient to the same cell, a micropipette filled with a mixture of cAMP and 0.1 g/mL Alexa 594 linked with a FemtoJet was positioned 1000 μm away from the cells and was then quickly moved to a position within 100 μm of the cells. During the experiments, we only changed the distance between the micropipette and the cells. The speed of movement determines how fast a stable gradient can form around a cell. Images were processed and analyzed by Zen 780 software (Zen Black). Images were further processed in Adobe Photoshop (Adobe Systems, San Jose, CA, USA), and the intensity of the ROI (region of interest) was explored and analyzed with Microsoft Office Excel (Redmond, WA, USA). To measure the membrane translocation of the indicated protein, we first measured the intensity change in the cytoplasm in response to uniformly applied stimuli or in the gradient over time. To obtain the relative intensity change in each individual cell during the time lapse, we divided its intensity at a given time (I_t_) by its intensity at time 0 (I_0_); consequently, the relative intensity of any cells at time 0 became 1. To compensate for significant photobleaching that occurs with long-time acquisition, we also normalized the intensity relative to the photobleaching of the cells. We then divided the normalized intensity at time 0 s (I_0_) by the normalized intensity at the given time (I_t_) to convert the normalized intensity change in the cytoplasm to membrane translocation. Lastly, we calculated and presented the mean and standard deviation (Mean ± SD) of peak membrane translocation from more than 5 independent cells.

### 2.3. Immunoblotting

Mouse monoclonal anti-GFP antibodies were purchased from Clontech Laboratories, Inc. (Mountain View, CA, USA). The anti-Gα2 rabbit antibody from Peter Devreotes, Johns Hopkins University. Anti-pan Ras mouse monoclonal antibody from EMD Millipore (Darmstadt, Germany) was used to detect *D. discoideum* Ras proteins. HRP-conjugated anti-mouse or anti-rabbit IgG was obtained from Jackson ImmunoResearch (West Grove, PA, USA).

### 2.4. Membrane Translocation Assay

The experiment was done as previously described [34].

### 2.5. Immunoprecipitation Assay

Differentiated C2GAP1-YFP expressing cells were suspended at 2 × 10^7^ with PM buffer with 2.5 mM caffeine and kept on ice before the assay. Cells were stimulated with 10 μM cAMP. 0.5 mL aliquots of cells at indicated time points were lysed with 10 mL immunoprecipitation buffer (IB, 20 mM Tris, pH8.0, 20 mM MgCl_2_, 10% glycerol, 2 mM Na_3_VO_4_, 0.25% NP40, and one tablet of Complete 1× EDTA-free proteinase inhibitor) for 30 min on ice. Cell extracts were centrifuged at 16,000× *g* for 10 min at 4 °C. Supernatant fractions were collected and incubated with 25 μL anti-GFP agarose beads (Proteintec, Rosemont, IL, USA) at 4 °C for 2 h. Beads were washed four times with immunoprecipitation buffer and proteins were eluted by boiling the beads in 50 μL SDS sample buffer (Bio-Rad, Hercules, CA, USA).

### 2.6. AlphaFold3-Assisted Analysis of Gα2-C2GAP1 Interaction Using NIH Biowulf Cluster

Protein–protein complex models were generated using AlphaFold3 version 3.0.1 (DeepMind, London, UK, 2024) installed as a singularity container with af3 wrapper script (https://github.com/NIH-HPC/af3) (accessed on 2 February 2026) on the NIH Biowulf high-performance computing cluster. Model parameters were downloaded from Google DeepMind with permission for non-commercial use. Protein sequences were obtained from dictyBase.org. Jobs were submitted using the default AlphaFold3 (AF3) settings. Predictions were generated for Gα2 (-GDP or -GTP bound), C2GAP1, Gβ, and Gγ along with six interactions, including Gα2GβGγ (-GDP or -GTP bound), C2GAP1-Gα2 (-GDP or -GTP bound), and Gα2GβGγ (-GDP or -GTP bound)-C2GAP1. Five predictions were generated for each job with the top-ranked structure saved in CIF and JSON format and then accessed and visualized in ChimeraX version 1.11.1. Model accuracy was annotated in ChimeraX using the AlphaFold palette, with color-scheme mapped to per-residue confidence scores (pLDDT) accessed from the B-factor column of each respective CIF file. The parameters of the binding affinity for predicted structures were calculated using PRODIGY based on instructions from https://github.com/haddocking/prodigy (accessed on 2 February 2026) [35].

## 3. Results

### 3.1. c2gapA^−^ Cells Fail to Exhibit Chemoattractant Concentration-Dependent Adaptation upon Exposure to a Steady Gradient

**Figure 1:**
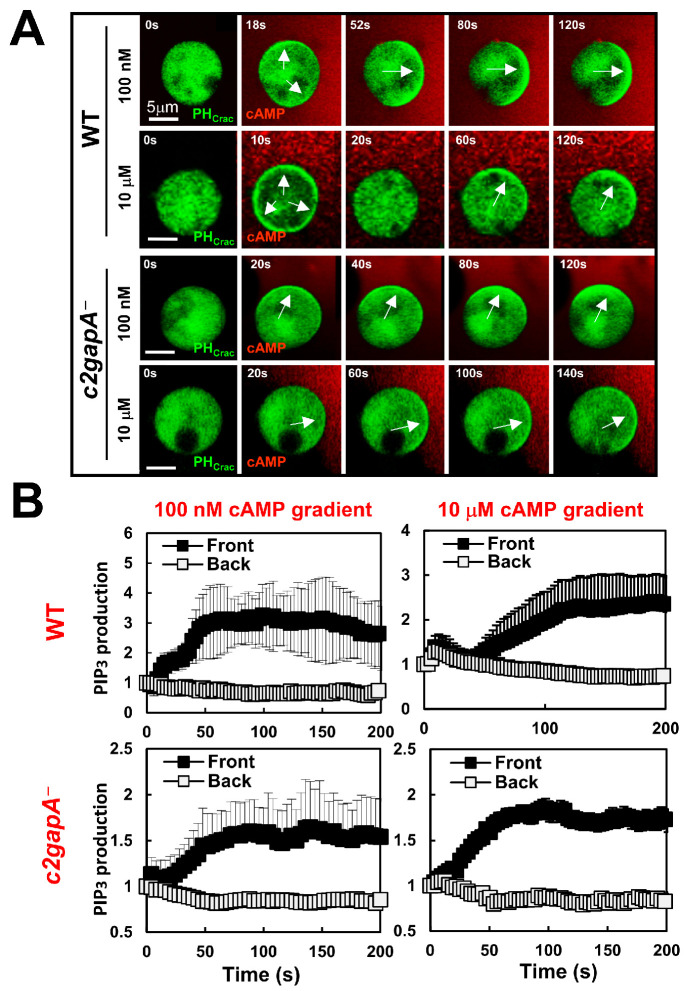
*c2gapA^−^* cells fail to display a concentration-dependent adaptive PIP_3_ dynamics of gradient sensing upon exposure to a steady gradient. (**A**). The montage shows PIP_3_ dynamics in wild-type (WT) and *c2gapA^−^* cells upon exposure to steady cAMP gradients generated from the sources of either 10 μM or 100 nM, respectively. Cells expressed PIP_3_ biosensor, PH_Crac_-GFP (green), were treated with 5 μM Latrunculin B for 10 min prior to the experiments. To visualize cAMP gradients, cAMP at the indicated concentrations was mixed with Alexa594 (red). See Video S1 for the complete set of cell responses. Scale bar = 5 μm. PH_Crac_-GFP translocation (PIP_3_ production) pointed out by arrows. (**B**). Normalized PIP_3_ production in the front and back of the cells exposed to steady gradients is shown. The PIP_3_ intensity in the front and region at time 0 s was normalized to 1. Mean ± SD is shown. *N* = 5 and 5 in WT and *c2gapA^−^* cells, respectively, at the indicated concentrations.

Chemotaxis is a coordinated process involving three key aspects: gradient sensing machinery, cell polarity, and cell migration. Gradient sensing can be uncoupled from initial cell polarity and cell migration [6]. For example, a cell treated with actin polymerization inhibitors loses its filamentous actin-based cytoskeleton and initial polarity, and subsequently becomes immobile, yet retains the ability to sense a chemoattractant gradient. This immobile cell provides a simplified experimental system to specifically investigate the gradient-sensing mechanisms. Using this approach, we previously determined the spatiotemporal dynamics of key signaling components during the polarization processes, including polarized PIP_3_ production/accumulation in the front of cells in response to exposure to a steady chemoattractant gradient [33]. A defining feature of gradient-sensing cells is their ability to efficiently establish intracellular polarity, manifested as a sharp accumulation of PIP_3_ in the front of cells toward the source of a chemoattractant, in a concentration-dependent fashion [33,36]. To understand the role of C2GAP1 in the adaptation process of gradient sensing, we first monitored PIP_3_ dynamics using a PIP_3_ biosensor, PH_Crac_-GFP, in both WT and *c2gapA^−^* cells exposed to cAMP gradients of varying concentrations (Figure 1A and Video S1). WT and *c2gapA^−^* cells expressing PH_Crac_-GFP (green) were treated with 5 μM Latrunculin B for 10 min prior to imaging to diminish cell polarity and cell migration. To visualize gradients, cAMP was mixed with Alexa594 (red) [37]. Upon exposure to a relatively low cAMP concentration gradient (100 nM), WT cells exhibited a single-phase PIP_3_ response, characterized by PH_Crac_-GFP translocation only to the front and lateral regions of cells, with minimal accumulation at the rear. The accumulation of PH_Crac_-GFP facing the gradient is also called a PH_Crac_ crescent [6]. In contrast, exposure to a high, saturating cAMP gradient (10 μM) induced a biphasic response in WT cells, consisting of an initial, uniform PH_Crac_-GFP translocation to the entire cell periphery, followed by withdrawal from the plasma membrane and a second, localized PH_Crac_-GFP accumulation (PH crescent, a PIP_3_ polarization) at the front, facing the gradient (Figure 1A, top panel). The observed termination of the initial PIP_3_ production reflects a typical adaptation process during gradient sensing and is consistent with previous reports [33,36]. We next examined PIP_3_ dynamics in *c2gapA^−^* cells exposed to either 100 nM or 10 μM cAMP gradients. In contrast to WT cells, *c2gapA^−^* cells displayed persistent PH_Crac_-GFP accumulations at the cell front under both concentrations, indicating a failure in adaptation during gradient sensing (Figure 1A, lower panel). The quantitative analysis of PIP_3_ dynamics during gradient sensing, assessed by measuring PH-GFP localization in the front and back (rear) regions of cells across multiple cells, further confirms this conclusion (Figure 1B). The above result demonstrates that C2GAP1 plays an essential role in PIP_3_ adaptation during gradient sensing in response to a gradient at high concentrations. 

### 3.2. c2gapA^−^ Cells Show an Altered PTEN Dynamics upon Exposure to a Steady Gradient

**Figure 2:**
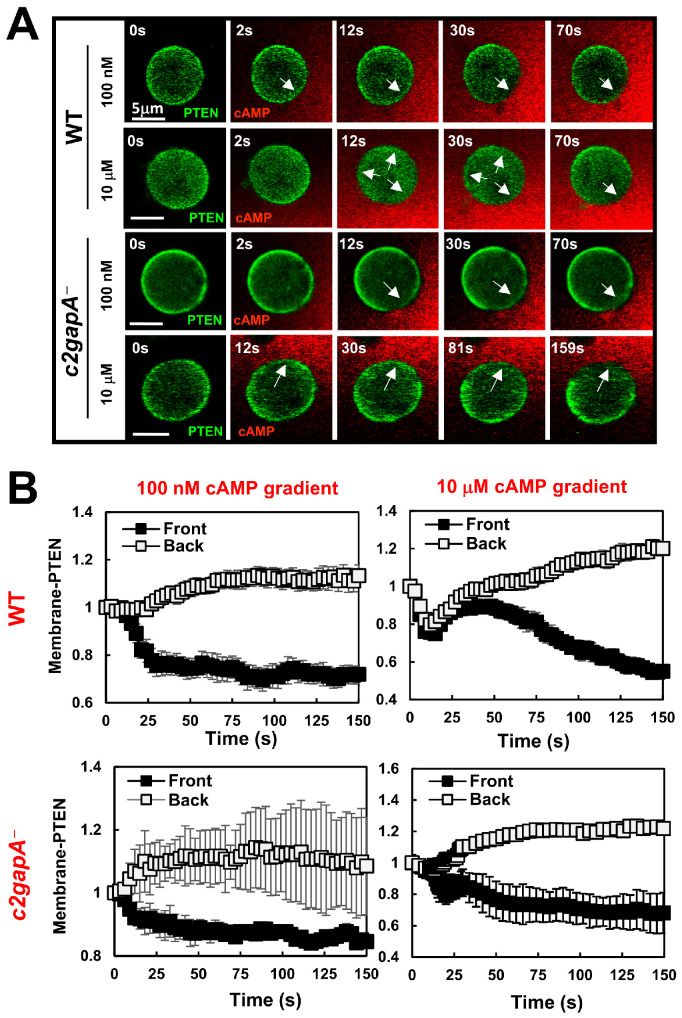
*c2gapA^−^* cells show altered PTEN dynamics upon exposure to a steady gradient. (**A**) The montage shows PTEN dynamics in WT and *c2gapA^−^* cells upon exposure to steady cAMP gradients at the indicated concentrations. Cells expressed PTEN-GFP (green) were treated with 5 μM Latrunculin B for 10 min prior to the experiments. To visualize cAMP gradients, cAMP at the indicated concentrations was mixed with Alexa594 (red). Scale bar = 5 μm. PTEN-GFP withdrawal from the plasma membrane pointed out by arrows. See Video S2 for complete sets of cell responses. (**B**) Normalized intensity of PTEN-GFP in the front and back of the cells upon exposure to steady gradients is shown. The PTEN intensity in the front and region at time 0 was normalized to 1. Mean ± SD is shown. *N* = 5 and 5 in WT and *c2gapA^−^* cells, respectively, at both indicated concentrations.

Phosphatase PTEN is a key molecule that dephosphorylates PIP_3_ and modulates PIP_3_ production during gradient sensing and chemotaxis [12,13]. We next monitored PTEN dynamics in WT and *c2gapA^−^* cells upon exposure to steady cAMP gradients at varying concentrations (Figure 2A and Video S2). WT and *c2gapA^−^* cells expressing PTEN-GFP (green) were treated with 5 μM Latrunculin B for 10 min prior to imaging. cAMP was mixed with Alexa594 (red) to visualize the gradient. PTEN-GFP localized to the plasma membrane in both unstimulated, resting WT and *c2gapA^−^* cells, as previously reported [13]. Upon exposure to a relatively low cAMP concentration gradient (100 nM), WT cells exhibited a single-phase PTEN response, characterized by withdrawal of PTEN-GFP from the front and lateral regions of cells (Figure 2A, upper panel). In contrast, exposure to a high, saturating cAMP gradient (10 μM) induced a biphasic PTEN response in WT cells, consisting of an initial, uniform withdrawal of PTEN-GFP from the entire cell periphery to the cytoplasm, followed by re-localization of PTEN-GFP from the cytoplasm to the rear of cells. This adaptive redistribution of PTEN restricts PIP3 accumulation to the cell front and is a hallmark of proper gradient sensing [33,36]. We next examined PTEN-GFP dynamics in *c2gapA^−^* cells exposed to either 100 nM or 10 μM cAMP gradients (Figure 2A, lower panel). In contrast to WT cells, *c2gapA^−^* cells displayed persistent withdrawal of PTEN-GFP at the cell front under both concentrations. This defective PTEN adaptation is consistent with the spatiotemporal PIP_3_ dynamics shown in Figure 1, resulting in sustained or excessive PIP_3_ accumulation at the leading edge. The quantitative analysis of PTEN dynamics during gradient sensing, assessed by PM-localization of PTEN-GFP in the front and back regions across multiple cells, further verifies the above conclusion (Figure 2B). In conclusion, the above results indicate that C2GAP1 plays an essential role in PTEN adaptative response during gradient sensing, especially in gradients at high concentrations.

### 3.3. F-Actin-Independent, cAMP Concentration-Dependent PM Targeting of C2GAP1 During Gradient Sensing

**Figure 3:**
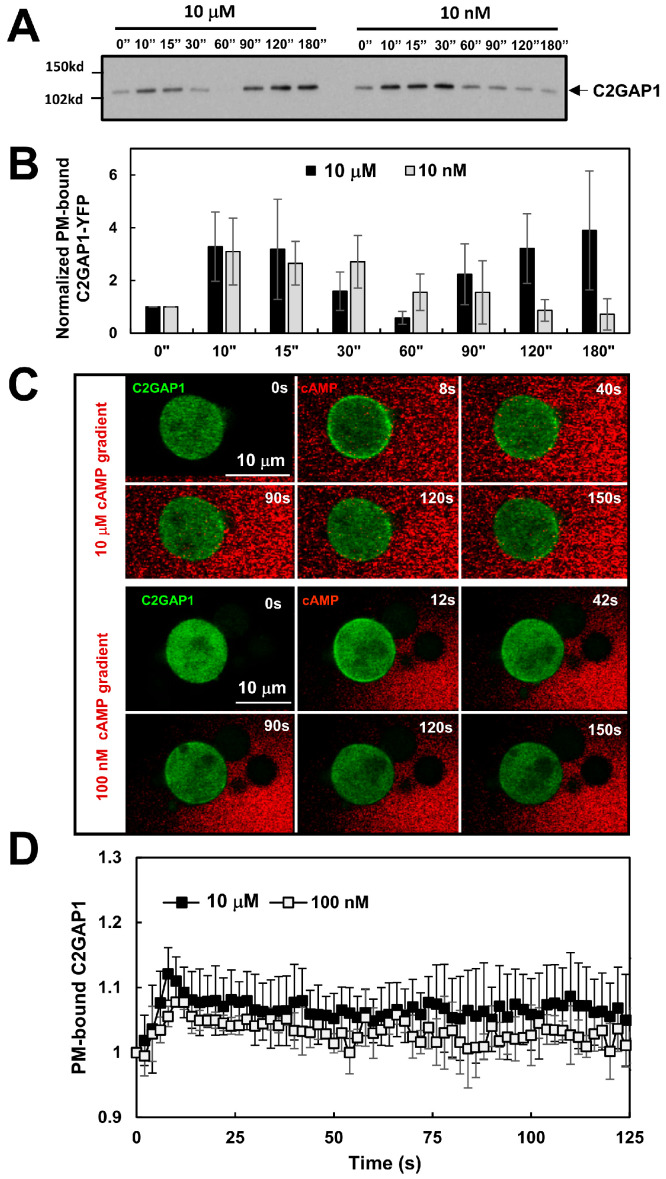
F-actin-independent, cAMP concentration-dependent PM targeting C2GAP1 during gradient sensing. (**A**) F-actin-independent, cAMP concentration-dependent plasma membrane translocation dynamics of C2GAP1 upon cAMP stimulation. C2GAP1-YFP expressing *c2gapA^−^* cells were treated with 5 μM Latrunculin B 10 min prior to the experiment and stimulated with cAMP at the indicated concentration at 0 s. Plasma membrane fractions were collected at the indicated time points and subjected to Western blot analysis to detect C2GAP1-YFP using anti-GFP antibodies. (**B**) Normalized plasma membrane (PM) translocation of C2GAP1 upon cAMP stimulation from (**A**) and two additional independent experiments (Figure S1). C2GAP1 membrane localization at 0 s was normalized to 1. (**C**) Montage shows C2GAP1-GFP dynamics in WT cells upon exposure to steady cAMP gradients at the indicated concentrations. Cells expressed C2GAP1-GFP (green) were treated with 5 μM Latrunculin B for 10 min prior to the experiments. To visualize cAMP gradients, cAMP at the indicated concentrations was mixed with Alexa594 (red). See Video S3 for complete sets of cell responses. (**D**) Normalized PM translocation of C2GAP1-GFP upon exposure to steady gradients is shown. The cytosolic intensity of C2GAP1 at time 0 s was normalized to 1. Mean ± SD is shown. *N* = 5 and 5 in WT cells exposed to cAMP gradients at either 10 μM or 100 nM, respectively.

To understand the F-actin-independent, chemoattractant concentration-dependent adaptation in gradient sensing, we next examined whether C2GAP1 translocates to the plasma membrane (PM) in an F-actin-independent, cAMP concentration-dependent manner. We first assessed PM translocation of C2GAP1–YFP biochemically in a large population of Latrunculin B-treated cells stimulated with either high (10 μM) or low (100 nM) cAMP. Plasma membrane fractions were isolated before and after cAMP stimulation as previously described [34]. Consistent with previous reports [31], we detected PM-bound C2GAP1 in resting, F-actin-free unstimulated cells and observed a clear cAMP concentration-dependent PM translation of C2GAP1-YFP (Figure 3A). Uniform stimulation at a high, saturating concentration of cAMP (10 μM) induced a biphasic PM translocation of C2GAP1: a rapid initial recruitment peaking at ~10–15 s, followed by dissociation from the PM by ~60 s, and subsequently a second, sustained PM localization lasting up to 180 s. In contrast, stimulation at a low concentration of cAMP (10 nM) elicited a single-phase response characterized by a slow but prolonged PM recruitment between ~10 and 30 s, followed by a return to pre-stimulation levels. Quantitative analysis of three independent experiments confirmed these observations (Figure 3B and Figure S1).

We next monitored the spatiotemporal PM translocation of C2GAP1-YFP in cells upon exposure to a steady cAMP gradient by confocal microscopy (Figure 3C and Video S3). *c2gapA^−^* cells expressing C2GAP1-YFP (green) were treated with 5 μM Latrunculin B for 10 min prior to imaging. cAMP was mixed with Alexa594 (red) to visualize the gradient. Consistent with the result shown in Figure 3A, C2GAP1-YFP displayed notable PM localization in resting, F-actin-free cells. Upon exposure to a high, saturating cAMP (10 μM) gradient, cells exhibited an initial PM translocation peak around ~ 8 s, followed by a withdrawal from the PM and were maintaining a sustained level on the PM (Figure 3C, upper panel). Notably, C2GAP1-YFP accumulated more prominently in the front than at the rear. Upon exposure to a low-concentration cAMP (100 nM) gradient, a similar response pattern was observed, although the peak translocation occurred more slowly at 12 s (Figure 3C, lower panel). Quantitative analysis of membrane translocation, assessed by cytosolic C2GAP1 depletion, confirmed these observations and further revealed a concentration-dependent response (Figure 3D). Collectively, these results demonstrate that C2GAP1 accumulates in the plasma membrane in an F-actin-independent, cAMP concentration-dependent manner during gradient sensing. Conclusively, C2GAP1 localizes on the plasma membrane in resting cells and chemoattractant gradients of both high and low concentrations trigger its translocation to the plasma membrane in an F-actin-independent manner. More importantly, the sustained plasma membrane localization of C2GAP1 following the initial translocation is concentration-dependent, being greater at the front than at the back of cells.

### 3.4. cAMP Concentration-Dependent Inhibitory Process of Gradient-Sensing Cells

**Figure 4:**
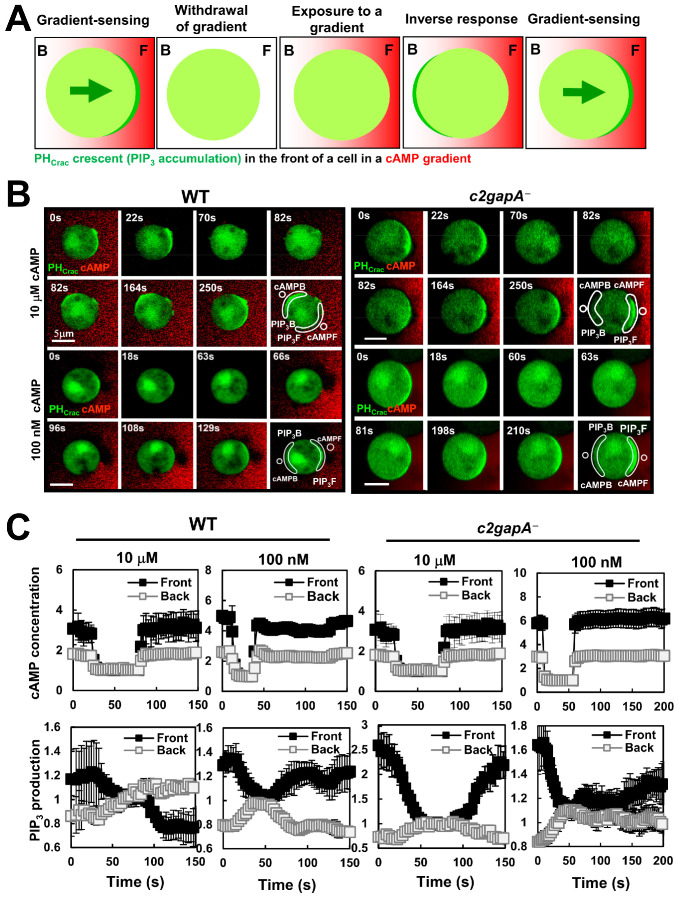
PIP_3_-polarized *c2gapA^−^* cells fail to display a concentration-dependent, higher inhibition in the front of gradient-sensing WT cells. (**A**) Scheme shows an inverse response of PIP_3_-polarized gradient-sensing cells upon removal and reapplication of the same gradient, whereby cells effectively experienced a higher stimulus at the front relative to the rear. (**B**) The montages show PIP_3_ dynamics in PIP_3_-polarized WT and *c2gapA^−^* cells upon removal of the original gradient and then re-exposure to a second, identical gradient at the indicated concentrations. Cells expressing PIP_3_ probe, PH_Crac_-GFP (green), were treated with 5 μM Latrunculin B for 10 min prior to the experiments. To visualize cAMP gradient, cAMP at the indicated concentrations was mixed with Alexa594 (Red). Gradient sensing capability was indicated by the accumulation of PH_Crac_-GFP in the front of the cells facing the source of a cAMP gradient as arrow shown. See Video S4 for complete sets of cell responses. (**C**) Normalized intensity of PIP_3_ and cAMP concentrations the front and back of *c2gapA^−^* cells upon removal and re-exposure to identical gradients. cAMP concentrations and PIP_3_ intensity at the time of complete withdrawal from plasma membrane upon the removal of the initial gradient was normalized to 1. Mean ± SD is shown. *N* = 5 and 9 in the gradients of either 10 μM or 100 nM cAMP, respectively.

One key feature of F-actin-free, PIP_3_-polarized gradient-sensing cells is that cells accumulate stronger inhibition in the front of cells facing a steady, persistent gradient [36,38]. This stronger inhibition was experimentally verified [38]. The experimental design is shown in Figure 4A. Cells initially exposed to a steady gradient displayed stable PH_Crac_-GFP crescents oriented toward the gradient source. Next, the gradient was removed, and the PH_Crac_-GFP crescents gradually diminished and disappeared in the front. Upon reapplication of a second stimulation (either a uniform or a gradient), PH_Crac_-GFP translocated exclusively at the original rear of the cells. We refer to this behavior as an inversed response. An inverse response was also elicited upon reapplication of the same gradient, whereby cells effectively experienced a higher stimulus at the front relative to the rear (Figure 4A). The eventual disappearance of the inversed response, or its reorientation and re-accumulation of PH_Crac_-GFP at the front facing the gradient source, demonstrates that cells retain their ability to sense and respond to the gradient [38]. We designated this increased sensitivity at the rear of PIP_3_-polarized cells as inverse sensitivity, reflecting the preferential responsiveness of the original back due to the higher inhibition in the front of gradient-sensing cells. To further elucidate the nature of inverse sensitivity, we investigated whether it depends on the cAMP concentration of the gradient. To address this, we investigated the inversed sensitivity of PIP_3_-polarized cells exposed to either high (10 μM) or low (100 nM) cAMP gradient. After cells established a stable PH_Crac_ crescent, the initial gradient was removed to allow dissipation of front-facing PH_Crac_-GFP accumulation. An identical, second gradient was reintroduced, and cellular response was monitored (Figure 4B, Video S4). We found that PIP_3_-polarized cells exposed to 10 μM cAMP gradients frequently (~93.6%) displayed inverse sensitivity (Figure 4B, left upper panel). In contrast, only ~8.2% of cells exposed to 100 nM cAMP gradients exhibited an inverse response (Figure 4B, left lower panel). The selected areas shown in the montages indicate the regions used for quantitative measurement of cAMP gradient sensing and PH_Crac_-GFP PM translocation. Quantitative analysis confirmed that identical gradients were applied to the cells and revealed the spatiotemporal dynamics of PIP_3_ accumulation at both the front and back of the cells (Figure 4C). Taken together, these results demonstrate that an F-actin-independent, cAMP concentration-dependent local inhibitory process is an intrinsic component of gradient sensing.

### 3.5. c2gapA^−^ Cell Fails to Display a Higher Inhibition in the Front During Gradient Sensing

C2GAP1 accumulates at the plasma membrane in a cAMP concentration-dependent manner, more at the front than at the back, in cells exposed to a cAMP gradient (Figure 3). We next investigated whether C2GAP1 contributes to the cAMP concentration-dependent inhibitory mechanism. To address this, we examined whether gradient-sensing, PIP_3_-polarized *c2gapA^−^* cells exhibit concentration-dependent inverse sensitivity. *c2gapA^−^* cells expressing the PIP_3_ biosensor PH_Crac_-GFP (green) were treated with 5 μM Latrunculin B for 10 min prior to the experiment and cAMP was mixed with Alexa594 (red). Upon exposure to either 10 μM or 100 nM cAMP gradients, *c2gapA^−^* cells displayed PH-GFP accumulation (PH crescents) at the front facing the gradient (Figure 4A, right panel; Video S4). Removal of either gradient caused PH_Crac_ crescents to gradually diminish and disappear. Upon re-exposure to the identical gradient, PH_Crac_-GFP translocated to the original front, instead of the back, of *c2gapA^−^* cells. The selected areas shown in the montages indicate the regions used for quantitative measurement of cAMP gradient sensing and PIP_3_ accumulation. Quantitative analysis confirmed that identical gradients were applied and revealed the spatiotemporal dynamics of PIP_3_ accumulation at both the front and back of the cells (Figure 4C). Collectively, these results demonstrate that C2GAP1 plays an essential role in establishing cAMP concentration-dependent, local inhibition during gradient sensing.

### 3.6. Dynamics of C2GAP1 PM Localization in Response to Removal and Second Application of cAMP Gradient

**Figure 5:**
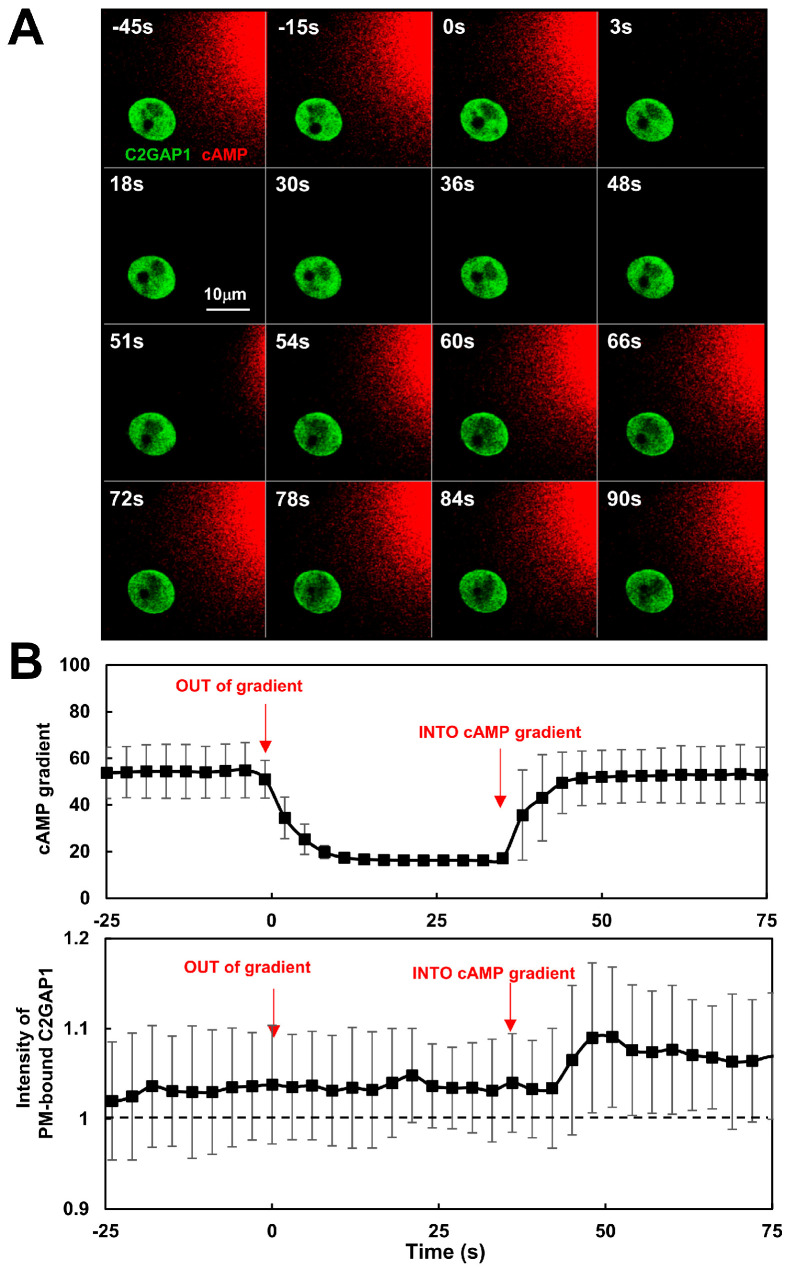
C2GAP1 dynamics in response to removal and second application of cAMP gradient. (**A**) The montages show C2GAP1-YFP (green) dynamics in PIP_3_-polarized WT and *c2gapA^−^* cells upon removal of the original gradient and then re-exposure to a second, identical gradient at the indicated concentrations. Cells expressing C2GAP1-YFP (green), were treated with 5 μM Latrunculin B for 10 min prior to the experiments. To visualize cAMP gradient, 10 μM cAMP was mixed with Alexa594 (red). See Video S5 for complete set of cell responses, including exposure to the initial gradient and then removal and reapplication of a second identical gradient. Time 0 s was set at the time last scan before the removal of the existing, original gradient and reapplication of the gradient at time 51s. (**B**) Normalized intensity of PM-bound C2GAP1 and cAMP concentrations in the front and back of *c2gapA^−^* cells upon removal and re-exposure to identical gradients. cAMP concentrations and PM-bound C2GAP1 intensity at the time of complete withdrawal from plasma membrane upon the removal of the initial gradient was normalized to 1. Mean ± SD is shown. *N* = 5.

To investigate the mechanism by which C2GAP1 mediates this concentration-dependent inhibition, we monitored plasma membrane localization of C2GAP1 following gradient removal and reapplication (Figure 5A; Video S5). Chemotactic C2GAP1-YFP (green)-expressing *c2gapA^−^* cells treated with latrunculin B (5 μM) were exposed to a 10 μM cAMP gradient (red). After exposure to a steady gradient for more than 200 s, the gradient was removed and this time point was designated as 0 s. At 54 s, an identical gradient was reintroduced, inducing a second translocation of C2GAP1-YFP to the plasma membrane followed by partial withdrawal. Quantitative analysis confirmed removal of the initial gradient and subsequent reapplication of an identical gradient (Figure 5B). Plasma membrane localization of C2GAP1 at the time of initial gradient exposure was normalized to 1; notably, membrane-bound C2GAP1 remained above this level at 0 s, indicating persistent plasma membrane association, consistent with Figure 3D. A complete dataset of gradient measurements and C2GAP1 PM translocation during initial exposure, gradient removal, and re-exposure is shown in Figure S2. Importantly, C2GAP1-YFP did not significantly dissociate from the plasma membrane following gradient removal, suggesting that C2GAP1 persists at the membrane to provide sustained inhibitory signaling during subsequent gradient sensing.

### 3.7. C2GAP1 Interacts with Gα2 to Decrease Cell Sensitivity and the Activation of Heterotrimeric G Protein upon cAMP Stimulation

**Figure 6:**
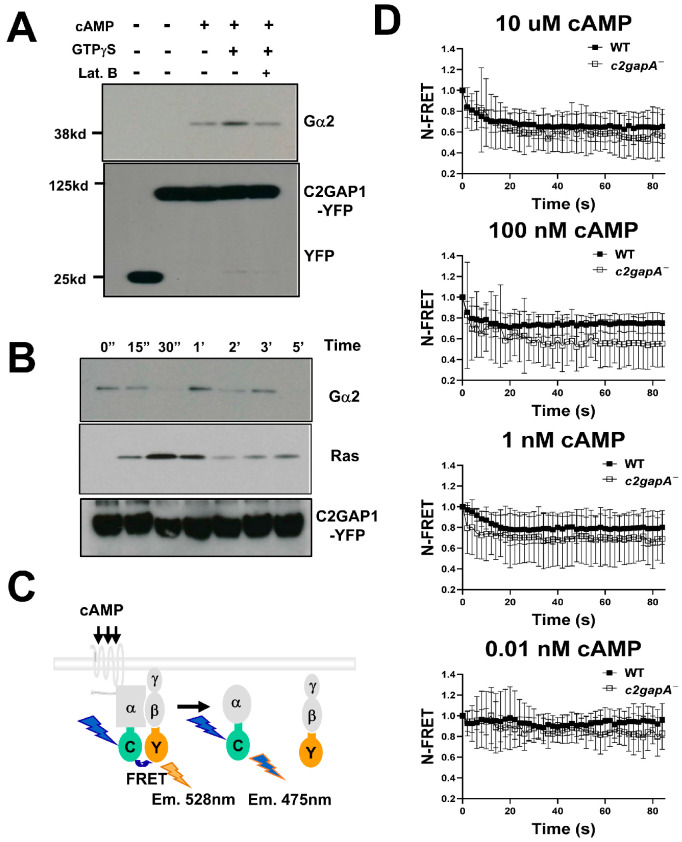
C2GAP1 interacts with Gα2 and decreases the activation of heterotrimeric G protein upon cAMP stimulation. (**A**) Interaction of C2GAP1 and Gα2 under various conditions determined by co-immunoprecipitation (co-IP). cAMP-chemotactic competent cells with endogenous level Gα2 and the expression of either YFP (a negative control) or C2GAP1-YFP treated with either 10 μM cAMP, 1 mM GTPγS, and/or 5 μM latrunculin B (Lat. B) were lysed and incubated with anti-GFP conjugated agarose beads and subjected to co-IP experiment and western-blotting detection of the indicated molecules. (**B**) Dynamic interaction between C2GAP1 and Gα2/Ras upon cAMP stimulation determined by co-immunoprecipitation (co-IP). cAMP-chemotactic competent cells with endogenous level Gα2 and the expression of C2GAP1-YFP were stimulated with cAMP at a final concentration of 10 μM. Aliquots of cells at the indicated time points were subject to co-IP experiment and western-blotting detection of the indicated molecules. C2GAP1-Ras interaction was used to as a positive control. (**C**) Schematic illustration of heterotrimeric G protein activation upon cAMP stimulation, measured as a loss of FRET between Gα2-CFP and Gβ-YFP. G protein activation leads to dissociation of Gα2 from Gβγ, resulting in reduced FRET efficiency. (**D**) Time course of FRET efficiency changes in WT and *c2gapA^−^* cells following uniform cAMP stimulation. FRET efficiency was quantified using sensitized emission-based FRET analysis.

To better understand the molecular mechanisms by which C2GAP1 modulates Ras signaling to establish a concentration-dependent inhibitory process to achieve the proper PIP_3_ dynamics, we sought to identify additional molecules that interact with C2GAP1 under different conditions by co-immunoprecipitation (co-IP) assays (Figure 6A). Cells expressing YFP protein alone were used as a negative control. cAMP-chemotactic C2GAP1-YFP-expressing cells were treated with either cAMP, hydrolysis-resistant GTPγS, or latrunculin B and then subjected to a co-IP experiment and immunoblotting of the indicated proteins. Notably, we identified Gα2 as an interacting partner of C2GAP1 with or without cAMP stimulation (Figure 6A). cAMP chemoattractant-competent cells express endogenous levels of Gα2 that is the α subunit of the heterotrimeric G protein essential for cAMP gradient sensing in F-actin-free cells [33,38]. Importantly, the association between Gα2 and C2GAP1 persisted in cells treated with an actin polymerization inhibitor, latrunculin B, when the F-actin-mediated positive or negative feedback loop was diminished, indicating that the Gα2-C2GAP1 interaction is a core component of the gradient-sensing machinery.

To further characterize the dynamics of the association, we determined the temporal profile of the C2GAP1-Gα2 interaction in response to cAMP stimulation (Figure 6B). The C2GAP1-Ras interaction was used as a positive control, and as previously reported, a transient association between C2GAP1 and Ras was observed [28]. Consistent with the result shown in Figure 6A, an association between Gα2 and C2GAP1 was present in the cells prior to stimulation. This interaction persisted but decreased markedly at 15 s after cAMP stimulation and became undetectable at 30 s, followed by a second peak at approximately 1 min post stimulation and subsequent oscillatory behavior. These interaction dynamics are consistent with previous observations that a fraction of C2GAP1 localizes to the plasma membrane and protrusive regions in resting cells, and that cAMP stimulation induces an initial plasma membrane translocation of C2GAP1 at ~15 s, followed by withdrawal at ~30 s, a second translocation at ~1 min, and subsequent oscillatory recruitment [28,31]. Notably, the second peak of the Gα2–C2GAP1 interaction coincided with the second wave of C2GAP1 plasma membrane localization during gradient sensing.

To assess the role of the Gα2-C2GAP1 interaction in heterotrimeric G protein activation, we measured G protein activation by monitoring fluorescence resonance energy transfer (FRET) between Gα2-CFP (FRET donor) and Gβ-YFP (FRET acceptor) in WT and *c2gapA^−^* cells by confocal live cell imaging as previously reported [33]. A schematic illustrating G protein activation upon cAMP stimulation, measured as a loss of FRET-between Gα2-CFP and Gβ-YFP, is shown in Figure 6C. Sensitized emission of FRET efficiency was used to quantify FRET loss [39]. In WT cells, cAMP stimulation at a final concentration of 10 μM induced a decrease in FRET efficiency (Figure 6D), consistent with previous reports [39,40]. *c2gapA^−^* cells exhibited a notably greater loss of FRET in response to the same 10 μM cAMP stimulation. This enhanced FRET loss was also observed in *c2gapA^−^* cells stimulated with lower concentrations of cAMP (100 nM and 1 nM). More importantly, *c2gapA^−^* cells responded to 0.01 nM cAMP stimulation, whereas WT cells no longer did. Together, these results demonstrate that the Gα2–C2GAP1 interaction not only sustains C2GAP1 at the plasma membrane to inhibit Ras signaling, thereby reducing cell sensitivity and promoting proper adaptation, but also attenuates heterotrimeric G protein activation in response to cAMP stimulation. Moreover, the C2GAP1–Gα2 interaction suppresses G protein activation in resting cells, further decreasing cellular sensitivity to chemoattractant stimulation.

### 3.8. Simulation of the Gα2-C2GAP1 Interaction

**Figure 7:**
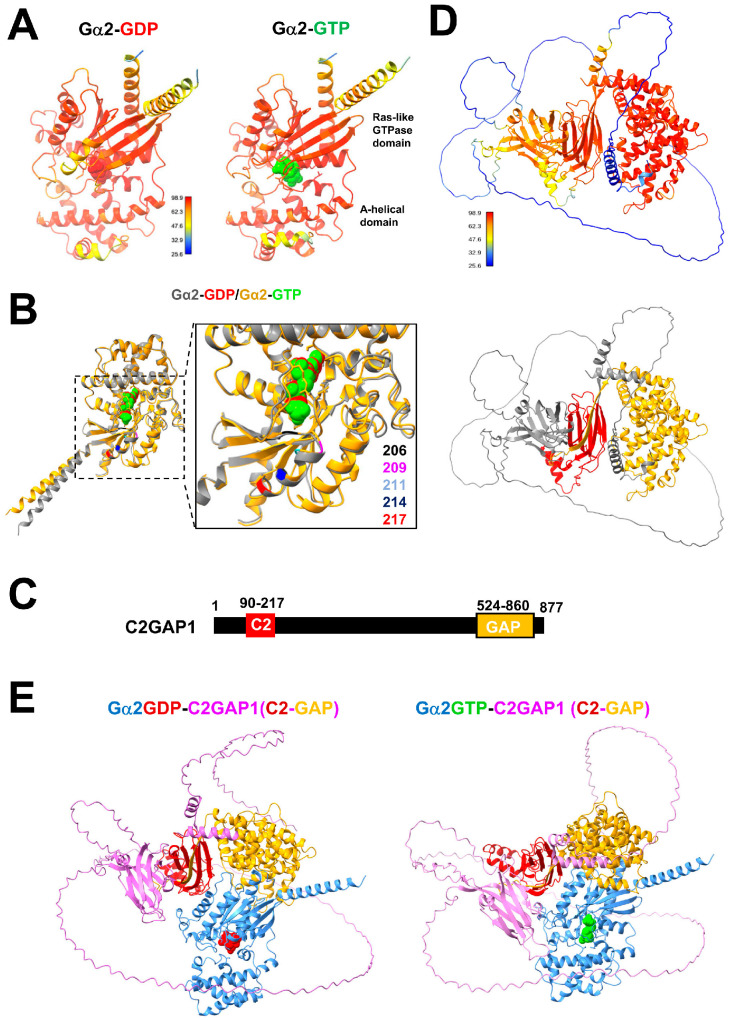
Simulation of the interaction between Gα2 and C2GAP1. (**A**) Predicted structures of the heterotrimeric Gα2 subunit in complex with GDP (red spheres) or GTP (green spheres). Gα2 is composed of a Ras-like GTPase domain and an α-helical domain, separated by a deep cleft that accommodates GDP or GTP. (**B**) Alignment of Gα2-GDP (gray) and Gα2-GTP (gold). The enlarged area highlights key residues in the switch region, with residue numbers displayed in matching colors. (**C**) Domain architecture of C2GAP1, comprising a C2 domain and a GAP domain. (**D**) Predicted structures of C2GAP1, with the prediction confidence scale shown in the upper panel and domain composition in the lower panel; the C2 domain is shown in red and the GAP domain in gold. (**E**) Predicted Gα2–C2GAP1 complexes, showing Gα2 (blue) in GDP-bound (red) or GTP-bound (green) states and the C2 (red) and GAP (gold) domains of C2GAP1 (magenta).

In resting cells, the majority of heteromeric G protein exists in the inactive GDP-bound form, Gα2(GDP)Gβγ. Saturating cAMP stimulation (10 μM cAMP; Figure 6B) induces maximal dissociation of the heterotrimer, leading to activation of Gα2-GTP and release of free Gβγ. Notably, Gα2–C2GAP1 association is detected both before and after cAMP stimulation, indicating that C2GAP1 can interact with Gα2 in either its inactive (GDP-bound) or active (GTP-bound) state. To gain mechanistic insight into this dynamic interaction, we modeled the structures and binding properties of Gα2 and C2GAP1 using AlphaFold 3 (Figure 7). We first predicted the structures of Gα2 in its GDP- and GTP-bound forms (Figure 7A). Gα2 adopts the canonical architecture composed of a Ras-like GTPase domain and an α-helical domain, separated by a deep cleft that accommodates GDP or GTP. The switch regions of Gα2 are similarly conserved (Figure 7B) [41]. These predicted structures show substantial overlap with mammalian Gαi2 (Figure S3A). We also modeled the Gβ and Gγ subunits: Gβ forms a seven-bladed β-propeller, with each blade consisting of four-stranded antiparallel β-sheets, whereas Gγ adopts a coiled-coil structure (Figure S3B), both closely resembling their mammalian counterparts (Figure S3C). The predicted heterotrimeric G protein complex (Gα2Gβγ) exhibits a conserved overall architecture relative to mammalian structures (Figure S3D) [42].

With these conserved structural features established for *Dictyostelium* heterotrimeric G proteins, we next predicted the structure of C2GAP1 (Figure 7C,D). The domain architecture for both the C2 and RasGAP domains was predicted with high confidence. We then modeled the complexes between C2GAP1 and Gα2 in either the GDP- or GTP-bound state (Figure 7E). We have previously shown that C2GAP1 binds multiple phospholipids on the plasma membrane through its C2 domain [31]. Surprisingly, both C2 and GAP domains interact with the Ras GTPase domain of Gα2 in both nucleotide states. Notably, an additional region of C2GAP1 located between the C2 and GAP domains provides extra contacts with the GTPase domain of Gα2 specifically in the GTP-bound state (Figure S4A). Binding free-energy calculations yielded predicted values of −9.8 kcal/mol for C2GAP1–Gα2-GDP and −11.2 kcal/mol for C2GAP1–Gα2-GTP, corresponding to inferred dissociation constants of ~6.8 × 10^−8^ M and ~6.3 × 10^−9^ M, respectively (Figure S4B). By nature of the simulations, these values should be interpreted qualitatively rather than quantitatively. Nevertheless, the results indicate an approximately one-order-of-magnitude stronger affinity of C2GAP1 for Gα2-GTP compared with Gα2-GDP. Together, these simulations support a model in which C2GAP1 associates with Gα2 in both its inactive and active states but preferentially binds activated Gα2-GTP. This enhanced interaction provides a molecular mechanism for the increased plasma membrane recruitment of C2GAP1 during cAMP gradient sensing.

### 3.9. c2gapA^−^ Cells Display Significantly Impaired Reorientation in Response to a Changing Gradient

**Figure 8:**
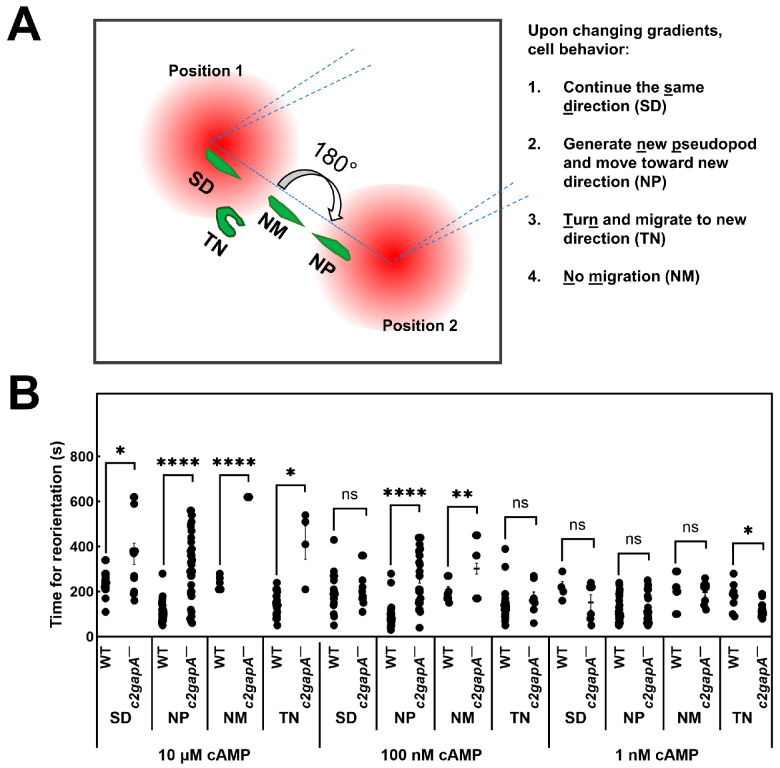
*c2gapA^−^* cell displays significant impaired reorientation in response to a changing gradient. (**A**) Schematic of the changing-direction experiment used to assess responses of WT and *c2gapA^−^* cells (see Video S6 for complete cell responses). Four distinct behaviors were observed: (1) continuous migration in the same direction (SD); (2) formation of a new pseudopod at the original trailing edge followed by migration toward the new gradient direction (NP); (3) turning of 180° and migrating toward the new gradient direction (TN); and (4) no movement (NM). (**B**) Time (s) required for reorientation in WT and *c2gapA^−^* cells upon the changing direction of the gradient. Sixty cells in each group from multiple-independent experiments were analyzed. Student *t* test was used to calculate the significance of difference, *p* > 0.05, ns (not significant); *p* < 0.05, *; *p* < 0.01, **; *p* < 0.0001, ****.

In the natural environment, chemoattractant gradients are often dynamic, and efficient reorientation is a key aspect of gradient sensing and subsequent chemotaxis. To investigate this, we designed an experiment to quantitatively measure the reorientation dynamics of both WT and *c2gapA^−^* cells in response to a changing gradient. Cells were first allowed to chemotax in a steady gradient for several minutes, after which the gradient was reversed, as illustrated in Figure 8A (see Video S6 for complete sets of cell responses). We observed four distinct cell behaviors: (1) continuous movement in the same direction (SD); (2) formation of a new pseudopod at the original trailing edge and migration toward the new gradient direction (NP); (3) turning of 180° and migrating toward the new gradient direction (TN); and (4) no movement (NM). We then quantitatively measured the time required for each of these behaviors in response to gradient changes at three different cAMP concentrations (10 μM, 100 nM, and 1 nM; Figure 8B). In 10 μM and 100 nM gradients, *c2gapA^−^* cells required significantly longer times to adjust to the new gradients compared with WT cells. This difference diminished at lower concentrations. At 1 nM, no significant difference was observed between WT and *c2gapA^−^* cells for SD, NP, or NM behaviors; however, *c2gapA^−^* cells displayed significantly faster turning and migration toward the new gradient direction. These results are consistent with previous reports showing that *c2gapA^−^* cells exhibit concentration-dependent chemotaxis: impaired chemotaxis in high, saturating gradients, similar performance at medium concentrations, and enhanced chemotaxis at low or sub-sensitive concentrations [28,31]. In conclusion, our data indicates that C2GAP1 is critical for rapid reorientation, particularly in gradients at medium to high concentrations.

## 4. Discussion

Eukaryotic cells sense and migrate through chemoattractant gradients with an enormous concentration range, such as 10^−5^ to 10^−9^ M cAMP in *D. discoideum* and 10^−5^ to 10^−9^ M SDF1a or fMLP in neutrophils. To migrate effectively across such broad concentration ranges, cells employ adaptation mechanisms, by which they adapt to the current stimulus while maintaining sensitivity to stronger signals, enabling continuous movement up a gradient. Chemotaxis involves three conceptually distinct yet interconnected processes: gradient sensing, cell polarity, and cell migration. Among these, gradient sensing provides the foundation for directional migration. Although many components acting through the F-actin-based cytoskeleton have been shown to play pivotal roles in chemotaxis, the core elements of the gradient-sensing machinery and the molecular mechanisms underlying adaptation are not fully understood. In this study, we identify a Gα2–C2GAP1 interaction and demonstrate its essential role in mediating adaptation during gradient sensing and in promoting efficient orientation in dynamic gradients.

The essence of gradient sensing is the ability to detect an extracellular gradient and establish an intracellular polarized response. Latrunculin B-treated, cytoskeleton-free, immobile *Dictyostelium* cells retain the capacity to sense gradients and therefore provide a simplified system for specifically investigating gradient sensing [6]. Using these immobile cells, the spatiotemporal dynamics of GPCR cAR1-mediated sequential signaling events—including extracellular gradient strength, heterotrimeric G protein activation, Ras activation, and PIP_3_ production—have been monitored upon exposure to a steady gradient [27,33,36,38] (Figure S5). Upon gradient exposure, cells experience higher chemoattractant concentrations at the front than at the back, triggering rapid and sustained G protein activation that is stronger at the front [33,36]. This observation indicates that adaptation occurs downstream of G protein activation and that G protein activation reflects the local chemoattractant concentration.

Upon exposure to a steady gradient, cells establish intracellular polarity, manifested by a sharp accumulation of PIP_3_ at the front of the cell facing the chemoattractant source in a concentration-dependent manner. When exposed to relatively low cAMP gradients (<100 nM), cells exhibit a single-phase PIP_3_ production and accumulation at the front and lateral regions, with minimal accumulation at the rear. In contrast, exposure to high, saturating cAMP gradients (>1 μM) elicits a biphasic PIP3 response, consisting of an initial uniform PIP_3_ production around the entire cell periphery, followed by its reduction from the plasma membrane and a second phase of PIP_3_ accumulation at the front—representing a typical adaptation process followed by a polarized response. Nevertheless, the polarized PIP_3_ response during gradient sensing is concentration-independent and serves as a hallmark of gradient sensing. PTEN is a lipid phosphatase that converts PIP_3_ to PIP_2_ and regulates PIP_3_ polarization [12,13]. The spatiotemporal dynamics of PTEN membrane localization are opposite to those of PIP_3_ in a concentration-dependent manner [36]. Upon exposure to low cAMP gradients (<100 nM), cells exhibit a single-phase withdrawal of PTEN from the front and lateral regions, with minimal withdrawal at the rear. In contrast, exposure to high cAMP gradients (>1 μM) elicits a biphasic PTEN response, consisting of an initial uniform withdrawal of PTEN from the entire cell periphery, followed by its return from the cytoplasm to the plasma membrane and then a second withdrawal from the front—representing a typical adaptation process followed by polarized PTEN localization, consistent with PIP_3_ dynamics upon exposure to a steady gradient. The concentration-dependent dynamics indicate that low-concentration gradients induce a balanced response, manifested as continuous accumulation of PIP3 or persistent withdrawal of PTEN at the cell front, thereby establishing intracellular polarity. In contrast, high-concentration gradients trigger excessively strong activation that must first be attenuated through adaptation, after which a polarized intracellular PIP3 or PTEN response is established via signal amplification. Consistent with this model, *c2gapA^−^* cells fail to exhibit adaptive PIP3 and PTEN responses when exposed to high-concentration gradients but show a normal single-phase response under low-concentration gradients (Figure 1 and Figure 2). These results indicate that C2GAP1 plays an essential role in terminating the initial response and enabling adaptation in cells exposed to high-concentration gradients. Interestingly, both low- and high-concentration gradients induce an initial plasma membrane (PM) translocation of C2GAP1, which gradually declines but remains persistently associated with the PM in a concentration-dependent manner (Figure 3). This residual PM-localized C2GAP1, together with the fraction that remains stably retained at later stages, collectively mediates the adaptive behavior during gradient sensing. Adaptation behaviors of *ddnf1^−^* and *c2gapA^−^* cells indicate that multiple RasGAP proteins are involved in adaptation during gradient sensing. It is important to investigate the PIP_3_ and PTEN dynamics in *ddnf1^−^* cells.

Ras activation, an upstream activator of PI_3_K that drives PIP_3_ production, represents the earliest GPCR-mediated signaling step in gradient sensing that exhibits adaptation [27]. Importantly, accumulation of active Ras at the front of gradient-sensing cells depends on gradient concentration [27,28]. In line with this, we observed concentration-dependent plasma membrane (PM) accumulation of C2GAP1 following the initial PM translocation in cells upon exposure to steady gradients to restrain Ras activity in the front (Figure 3). Consistent with preferential C2GAP1 accumulation at the front relative to the back (Figure 3), we previously reported temporally stronger inhibition in the front of PIP_3_-polarized cells in gradients [38], indicating the existence of a local inhibition process during gradient sensing. Notably, this enhanced inhibition was observed only in cells experiencing strong, high-concentration gradients, but not low-concentration gradients (Figure 4). In contrast, *c2gapA^−^* cells failed to exhibit this stronger inhibition in PIP_3_-polarized cells at any gradient concentration, demonstrating an essential role for C2GAP1 in establishing enhanced inhibition in the front during gradient sensing. In agreement with the above, *c2gapA^−^* cells display excessive actin polymerization, subsequently broadening the leading edge during chemotaxis when experiencing a high-concentration gradient, demonstrating the necessity of C2GAP1 accumulation in the leading edge to tune down Ras signaling for proper polarization and efficient cell migration during chemotaxis [28]. Interestingly, membrane targeting of C2GAP1 does not closely correlate with the distribution of either active Ras or multiple phospholipids that bind to the C2 domain of C2GAP1 on the plasma membrane [28,31] (Figure 6B), suggesting the involvement of additional binding partners in C2GAP1 PM localization. In this study, we identify Gα2 as a plasma membrane binding partner of C2GAP1 in an F-actin-independent manner to build the local inhibition in gradient sensing cells (Figure 6A). Consistent with our report, it has recently been reported that Ras suppression potentiates rear actomyosin contractility-driven cell polarization and migration [43]. Simulations of binding affinity between C2GAP1 and Gα2 in either the GDP- or GTP-bound state suggest stronger interaction with activated Gα2, providing a mechanism by which Gα2 sustains C2GAP1 on the plasma membrane (Figure 7). Moreover, we detected enhanced G protein activation in *c2gapA^−^* cells (Figure 6D), demonstrating the inhibitory role of the C2GAP1–Gα2 interaction in G protein activation upon stimulation. This suppressive role of the C2GAP1–Gα2 interaction in regulating G protein activation is essential for rapid reorientation in dynamically changing gradients (Figure 8), exemplifying an additional layer of G protein regulation during gradient sensing. Investigating G protein activation under these experimental conditions remains challenging, but it may ultimately provide a definitive answer.

Active Ras has served as the hallmark of basal sensitivity and activity of cells. In both *Dictyostelium* and mammalian neutrophils, multiple RasGAP proteins have been reported to play a role in basal activity and cell migration [28,31,34,40,43,44]. Plasma membrane and pseudopod localization of these RasGAPs in resting and migrating cells further verify the deactivating role of Ras to tune down basal Ras activity and cell migration. Both *Dictyostelium* and human neutrophils deficient of RasGAPs or with hyper Ras activity are more sensitive and display improved chemotaxis in gradients at low or subsensitive concentrations, while they exhibit impaired chemotaxis in gradients at high, saturating concentrations [28,31,40,45]. The above phenomenon indicates an upshifted chemoattractant concentration range for efficient chemotaxis in RasGAP-deficient or Ras-hyperactive cells [46]. In the present study, we found that C2GAP1 directly interacts with both GDP- or GTP-bound Gα2 (Figure 6). *c2gapA^−^* cells display clear G protein activation to a subsensitive, 0.01 nM cAMP simulation, demonstrating the inhibitory function of C2GAP1 and Gα2, providing an upstream module to regulate cell sensitivity.

## Data Availability

The original contributions presented in this study are included in the article/Supplementary Material. Further inquiries can be directed to the corresponding author.

## Supplementary Materials

The following supporting information can be downloaded at: https://www.mdpi.com/article/10.3390/cells15090819/s1, Figure S1: Dose-dependent PM targeting C2GAP1 in the cells; Figure S2: Dynamics of C2GAP1 in response to application, removal, and reapplication of identical steady gradients; Figure S3: Predicted structures of heterotrimeric G protein Gα2Gβγ; Figure S4: Interfaces of Gα2−C2GAP1 interaction; Figure S5: Distinct dynamics of GPCR cAR1-mediated signaling during gradient sensing in cytoskeleton-free, immobile cells; Video S1: PIP_3_ dynamics of gradient sensing in WT (top) or *c2gapA^−^* (bottom) upon exposure to steady cAMP gradient generated from the source of 100 nM (left) or 10 μM (right) cAMP, respectively. Cells expressing PIP_3_ biosensor PH_Crac_-GFP (green) were treated with 5 μM latrunculin B 10 min prior to the experiment. To visualize cAMP gradient, cAMP was mixed with fluorescent dye Alexa 594 (red); Video S2: PTEN dynamics of gradient sensing in WT (top) or *c2gapA^−^* (bottom) upon exposure to steady cAMP gradient generated from the source of 100 nM (left) or 10 μM (right) cAMP, respectively. Cells expressing PTEN-GFP (green) were treated with 5 μM latrunculin B for 10 min prior to the experiment. To visualize cAMP gradient, cAMP was mixed with fluorescent dye Alexa 594 (red); Video S3: C2GAP1-GFP dynamics of gradient sensing in *c2gapA^−^* upon exposure to steady cAMP gradients generated from the source of either 10 μM (left) or 100 nM (right) cAMP, respectively. Cells expressing C2GAP1-YFP (green) were treated with 5 μM latrunculin B for 10 min prior to the experiment. To visualize cAMP gradient, cAMP was mixed with fluorescent dye Alexa 594 (red); Video S4: PIP_3_ dynamics of gradient sensing in WT (top) and *c2gapA^−^* (bottom) cells upon exposure to withdrawal and reapplication of the same cAMP gradient generated from the source of either 10 μM (left) or 100 nM (right) cAMP, respectively. Cells expressing PIP_3_ biosensor, PH_Crac_-GFP (green) were treated with 5 μM lat. B 10 min prior to the experiment. To visualize cAMP gradient, cAMP was mixed with fluorescent dye Alexa 594 (red); Video S5: C2GAP1-GFP dynamics of gradient sensing in *c2gapA^−^* cells upon exposure to withdrawal and reapplication of the same cAMP gradient generated from the source of 10 μM cAMP. Cells expressing C2GAP1-YFP (green) were treated with 5 μM latrunculin B for 10 min prior to the experiment. To visualize cAMP gradient, cAMP was mixed with fluorescent dye Alexa 594 (red); Video S6: Migration behavior of WT (left) and *c2gapA^−^* (right) cells in the gradients generated from the source of either 10 μM (top), 100 nM (middle), or 1 nM (bottom) cAMP, respectively.
