# Supplementary material for: Heterotrimeric G Protein–RasGAP Coupling Drives Adaptation During Chemotaxis
Source: Cells. 2026 Apr 30;15(9):819. doi: 10.3390/cells15090819 (PMC13163001; doi:10.3390/cells15090819)
Supplement: Supplementary file 1 [file cells-15-00819-s001.zip › cells-4195709-supplementaryupdate/Supplementary information_Final.pdf]

Supplementary information:

Supplemental figure legends:

Figure S1. **Dose-dependent PM targeting C2GAP1 in the cells.** Two independent measurements are shown in **A** and **B** for the quantification of C2GAP1 PM targeting shown in **Figure 3B**.

Figure S2. **Dynamics of C2GAP1 in response to application, removal, and reapplication of identical steady gradients.**

A. cAMP concentrations during gradient application, removal, and reapplication. cAMP levels were quantified by measuring the fluorescence intensity of Alexa594 surrounding cells; Alexa594 was mixed with cAMP to visualize the gradient.

B. Plasma membrane (PM)–associated C2GAP1-YFP in *c2gapA*$^{-}$ cells during gradient application, removal, and reapplication. PM-bound C2GAP1-YFP was quantified as depletion of C2GAP1-YFP from the cytoplasm and normalized to 1 at 0 s. Data are presented as mean ± SD. $N = 5$ and 9 for gradients of 10 μM and 100 nM cAMP, respectively.

Figure S3 **Predicted structures of heterotrimeric G protein Gα2Gβγ.**

A. Structural alignment of *Dictyostelium* Gα2 (gold) and human Gαi2 (cyan) in either GDP-bound (red) or GTP (green)-bound state.

B. Predicted structures of *Dictyostelium* Gβ and Gγ with prediction confidence shown by the confidence scale.

C. Structural alignment of *Dictyostelium* Gβ (blue) and mammalian Gβ1 (grey), and *Dictyostelium* Gγ (blue) and mammalian Gγ (grey).

D. Predicted heterotrimeric G protein complex of *D. discoideum* Gα2Gβγ with Gα2 in either the GDP- or GTP-bound state. Gα2 is shown in grey, Gβ in yellow, and Gγ in blue. GDP and GTP are shown in red and Green, respectively.

Figure S4. **Interfaces of Gα2–C2GAP1 interaction.**

A. Predicted Gα2–C2GAP1 complexes, showing Gα2 (blue) in GDP-bound (red) or GTP-bound (green) states and the C2 (red) and GAP (gold) domains of C2GAP1 (magenta).

B. Predicted parameters of binding affinity between (GDP- or GTP-bound) Gα2 and C2GAP1 interaction.

Figure S5. **Distinct dynamics of GPCR cAR1–mediated signaling during gradient sensing in cytoskeleton-free, immobile cells.**

A. Four cAR1-mediated signaling events activated by a cAMP gradient during gradient sensing.

B. chematic illustrating the front and back regions of a cell, where the signaling dynamics in response to a steady cAMP gradient are quantified in **C**.

C. Distinct temporal dynamics of G protein activation and PIP_3 production (reflecting PI_3K activation) at the cell front (solid lines) and back (dashed lines) following exposure to either low- or high-concentration cAMP gradients.

Supplemental videos

Video S1. PIP_3 dynamics of gradient sensing in WT (top) or *c2gapA*⁻ (bottom) upon exposure to steady cAMP gradient generated from the source of 100 nM (left) or 10 µM (right) cAMP, respectively. Cells expressing PIP_3 biosensor PH_{Crac}-GFP (green) were treated with 5 µM latrunculin B 10 min prior to the experiment. To visualize cAMP gradient, cAMP was mixed with fluorescent dye Alexa 594 (red).

Video S2. PTEN dynamics of gradient sensing in WT (top) or *c2gapA*⁻ (bottom) upon exposure to steady cAMP gradient generated from the source of 100 nM (left) or 10 µM (right) cAMP, respectively. Cells expressing PTEN-GFP (green) were treated with 5 µM latrunculin B for 10 min prior to the experiment. To visualize cAMP gradient, cAMP was mixed with fluorescent dye Alexa 594 (red).

Video S3. C2GAP1-GFP dynamics of gradient sensing in *c2gapA*⁻ upon exposure to steady cAMP gradients generated from the source of either 10 µM (left) or 100 nM (right) cAMP, respectively. Cells expressing C2GAP1-YFP (green) were treated with 5 µM latrunculin B for 10 min prior to the experiment. To visualize cAMP gradient, cAMP was mixed with fluorescent dye Alexa 594 (red).

Video S4. PIP_3 dynamics of gradient sensing in WT (top) and *c2gapA*⁻ (bottom) cells upon exposure to withdrawal and reapplication of the same cAMP gradient generated from the source of either 10 µM (left) or 100 nM (right) cAMP, respectively. Cells expressing PIP_3 biosensor, PH_{Crac}-GFP (green) were treated with 5 µM lat. B 10 min prior to the experiment. To visualize cAMP gradient, cAMP was mixed with fluorescent dye Alexa 594 (red).

Video S5. C2GAP1-GFP dynamics of gradient sensing in *c2gapA*⁻ cells upon exposure to withdrawal and reapplication of the same cAMP gradient generated from the source of 10 µM cAMP. Cells expressing C2GAP1-YFP (green) were treated with 5 µM latrunculin B for 10 min prior to the experiment. To visualize cAMP gradient, cAMP was mixed with fluorescent dye Alexa 594 (red).

Video S6. Migration behavior of WT (left) and *c2gapA*$^{-}$ (right) cells in the gradients generated from the source of either 10 μM (top), 100 nM (middle), or 1 nM (bottom) cAMP, respectively.

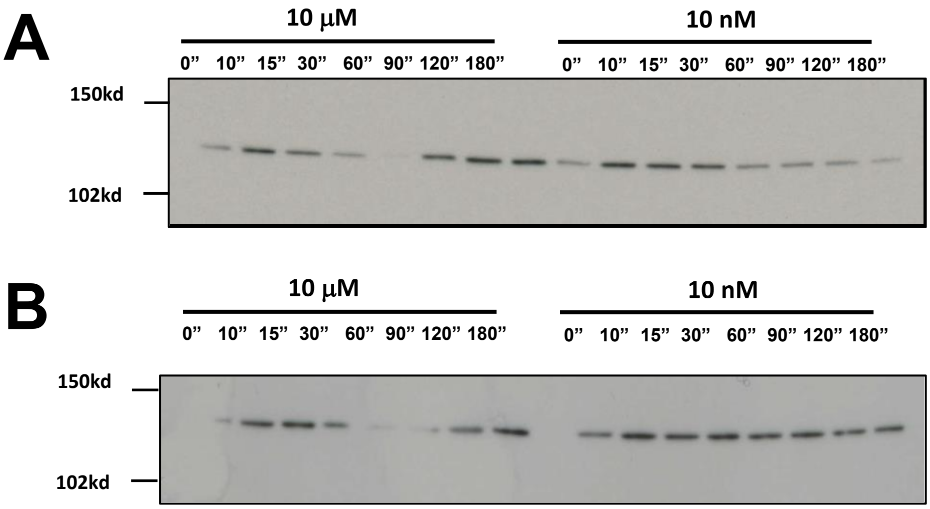
A
10 μM
10 nM
0” 10” 15” 30” 60” 90” 120” 180”
0” 10” 15” 30” 60” 90” 120” 180”
150kd
102kd
B
10 μM
10 nM
0” 10” 15” 30” 60” 90” 120” 180”
0” 10” 15” 30” 60” 90” 120” 180”
150kd
102kd

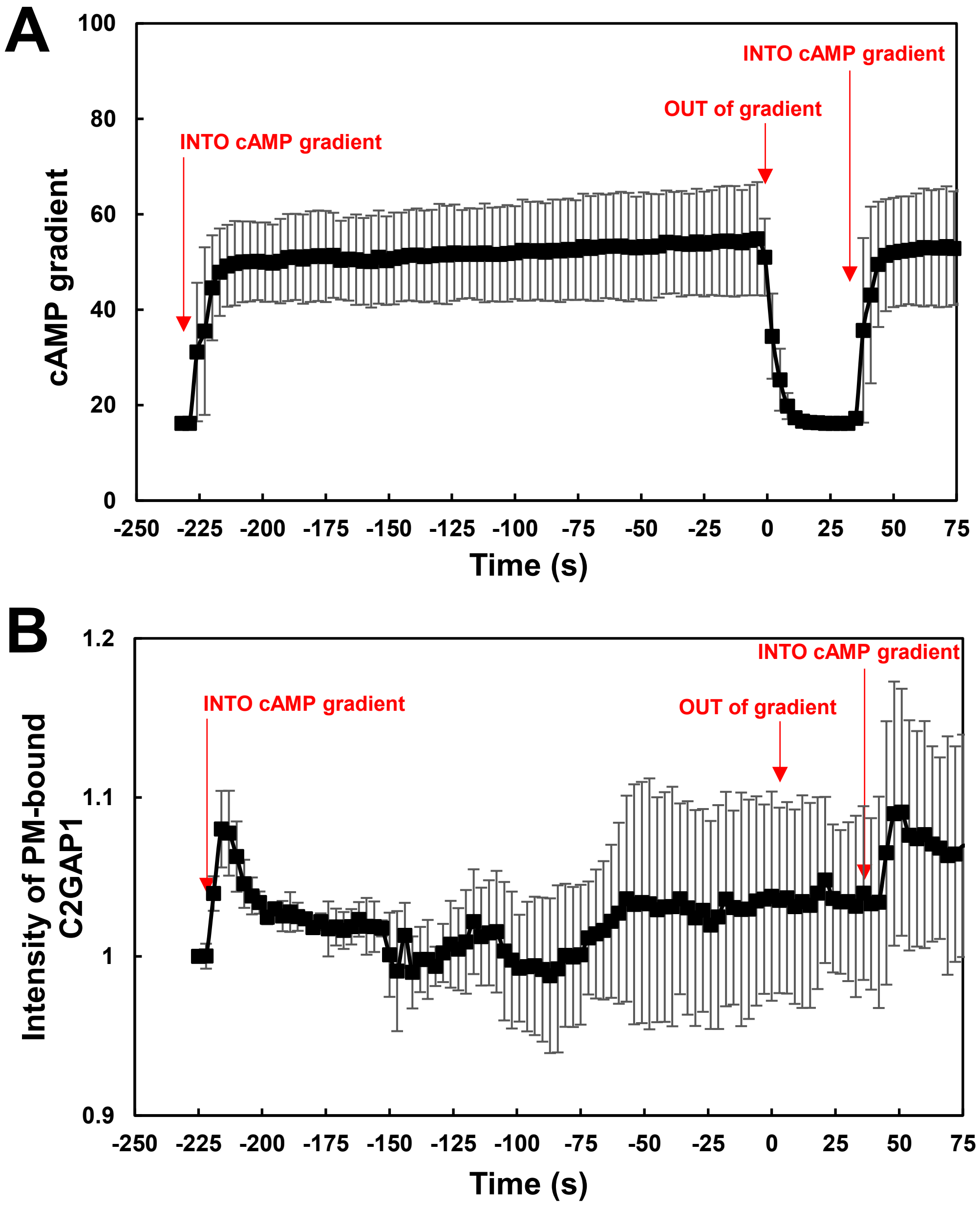
A
100
80
60
40
20
0
cAMP gradient
INTO cAMP gradient
OUT of gradient
INTO cAMP gradient
-250
-225
-200
-175
-150
-125
-100
-75
-50
-25
0
25
50
75
Time (s)
B
1.2
1.1
1
0.9
Intensity of PM-bound C2GAP1
INTO cAMP gradient
OUT of gradient
INTO cAMP gradient
-250
-225
-200
-175
-150
-125
-100
-75
-50
-25
0
25
50
75
Time (s)

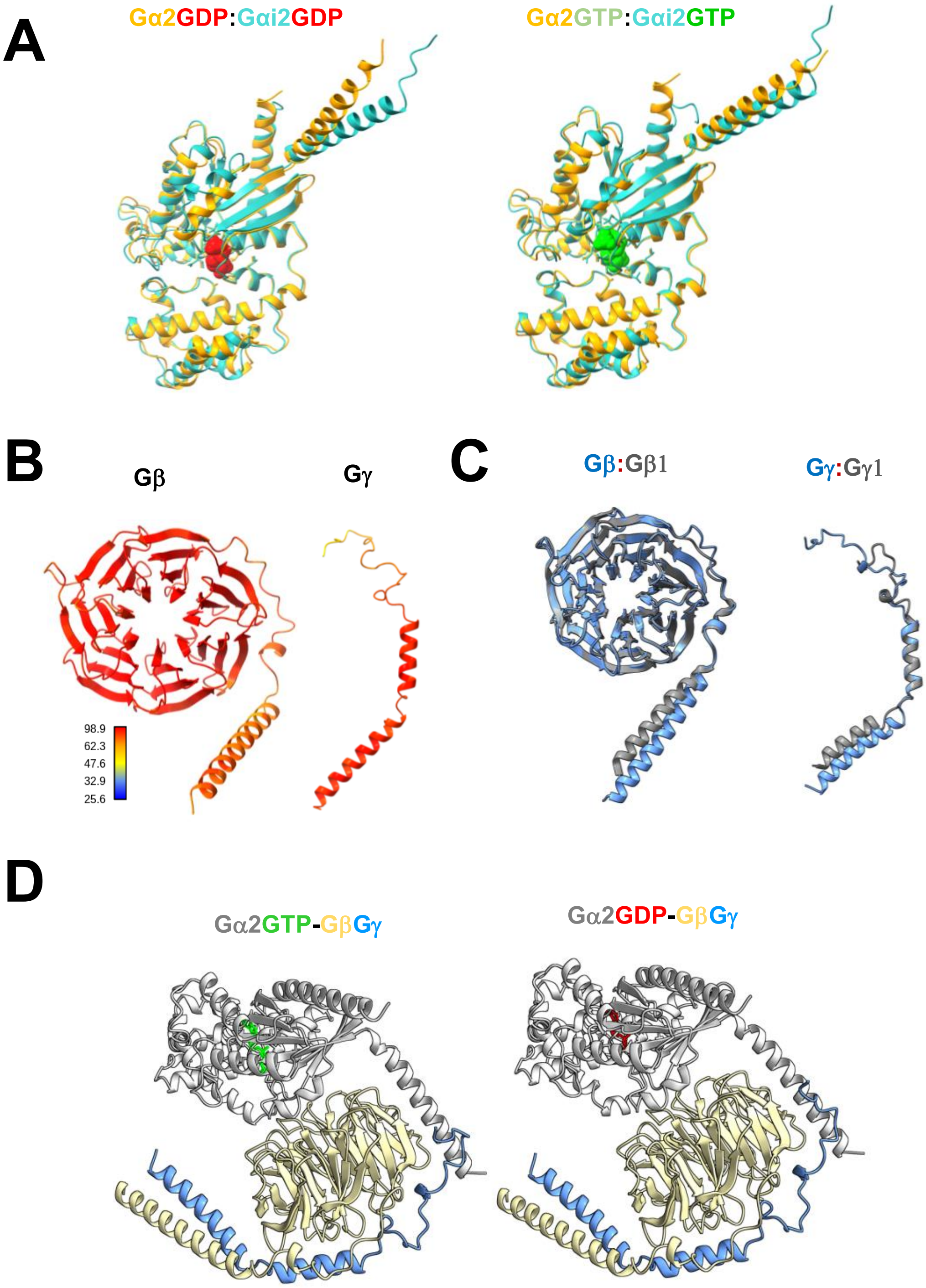
A
Gα2GDP:Gαi2GDP
Gα2GTP:Gαi2GTP
B
Gβ
Gγ
98.9
62.3
47.6
32.9
25.6
C
Gβ:Gβ1
Gγ:Gγ1
D
Gα2GTP-GβGγ
Gα2GDP-GβGγ

A

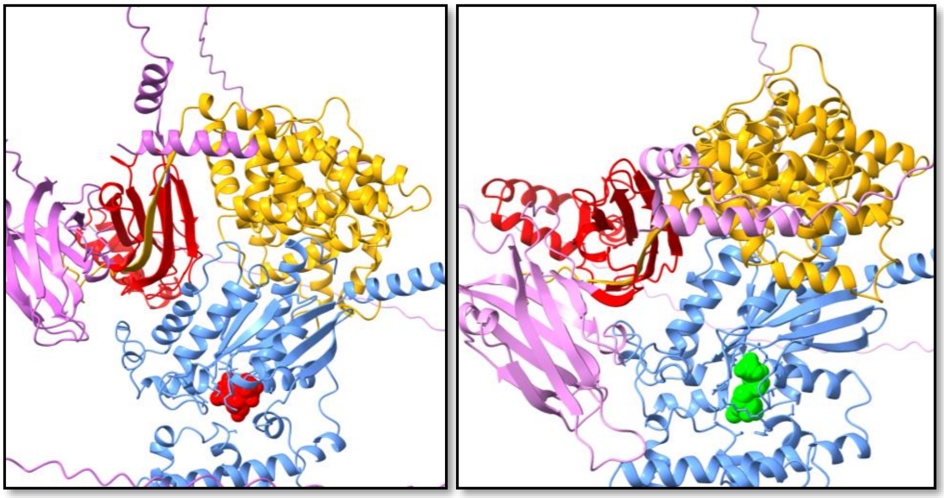

B

	C2GAP1/Gα2-GDP	C2GAP1/Gα2- GTP
# of intermolecular contacts:	62	73
# of charged contacts:	14	14
# of charged-polar contacts:	11	10
# of charged-apolar contacts:	17	27
# of polar-polar contacts:	3	1
# of apolar-polar contacts:	6	6
# of apolar-apolar contacts:	11	15
% of apolar NIS residues:	32.21	31.90
% of charged NIS residues:	28.63	28.90
Predicted binding affinity (kcal/mol):	-9.8	-11.2
Predicated dissociation constant:	6.8e-08	6.3e-09

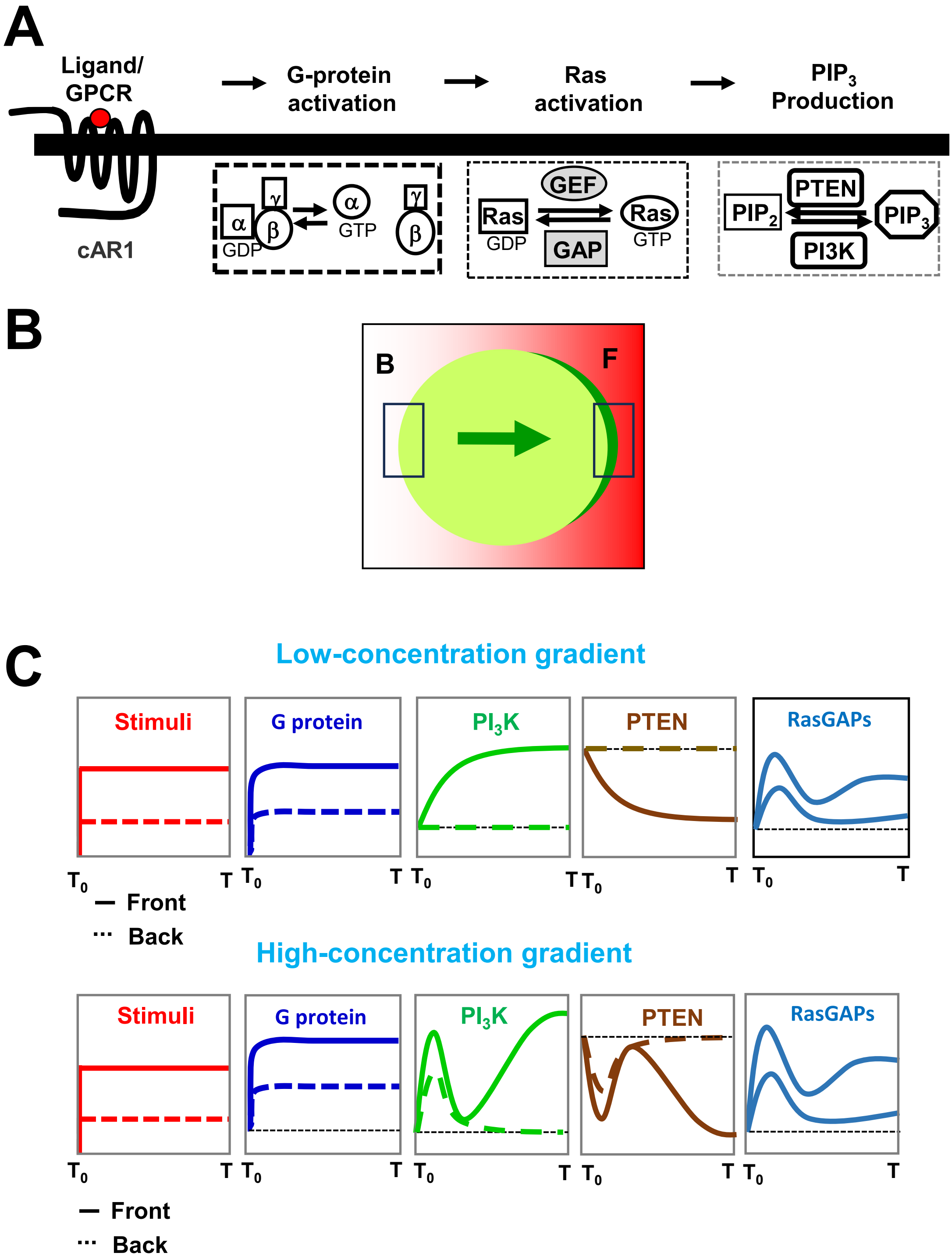
A
Ligand/
GPCR
G-protein
activation
Ras
activation
PIP3
Production
cAR1
α
β
γ
GDP
α
GTP
γ
β
GEF
Ras
GDP
Ras
GTP
GAP
PTEN
PIP2
PIP3
PI3K
B
B
F
C
Low-concentration gradient
Stimuli
G protein
PI3K
PTEN
RasGAPs
T0
T
Front
Back
High-concentration gradient
Stimuli
G protein
PI3K
PTEN
RasGAPs
T0
T
Front
Back

Graphic abstract:
